# Cracking and damage analysis of a masonry structure based on joint masonry model caused by a obliquely undercrossing tunnel

**DOI:** 10.1038/s41598-023-42399-1

**Published:** 2023-09-25

**Authors:** Junwei Jin, Qinglong Zhang, Boyi Fu, Jian Chen, Mingyu Li, Qianqian Jin

**Affiliations:** 1 School of Civil Engineering, Zhengzhou University, Zhengzhou, 450001 China; 2 China Railway Construction Underwater Tunnel Engineering Laboratory, Jinan, 250101 China; 3 School of Civil Engineering, Southwest Jiaotong University, Chengdu, 610031 China; 4 China Railway 14th Bureau Group Co., Ltd., Jinan, 250101 China

**Keywords:** Civil engineering, Environmental impact

## Abstract

The damage variation of a masonry structure during shield tunneling has been investigated. Furthermore, the damage degree of the masonry building has been investigated by combining the field measurement, finite element method results, and theoretical method. The results show that compared with the theoretical calculation method LSTM, the method provide by this paper can give the detail damage location of the masonry structure caused by shield construction. Due to the existence of door and window openings, the result of JMM is larger than LSTM result, the differences can be modified by concept of “characteristic tensile strain”. The wall of the masonry building encounter the shield face first suffers more damage than the later ones. At the beginning of tunnlling, the damage were generated from a small area at the bottom for wall 1, but a larger area at the top of the building for wall 2. The damage area increase more at the top of the building as tunneling advanced, but the maximum damage occurred at the bottom of the building.

## Introduction

New subways have been constructed mainly in urbans area nowadays. Consequently, in some cases, new tunnels must under-pass existing buildings. Shield tunnel construction inevitably disturbs the surrounding soil and causes displacement in different dimensions^1^. When buildings are located near a tunnel, settlement and deformation problems will emerge^2^. However, many masonry structures in urban centers of China are weak and not flexible. Therefore, accurately predicting the damage to existing buildings before constructing the shield tunnel is necessary.

The first analysis step is to determine the relationship between the building and the tunneling direction. Different tunneling directions imply that the building is in a separate settlement trough, and other parts of the building are affected differently. Most studies focused on the orthogonal distribution between the tunneling direction and building and only examined the influence of the deviation between the tunnel axis and building center^3,4^. The equivalent elastic beam method has been widely used to examine the soil–structure interaction due to the deformation of masonry structures dominated by wall behavior^5–7^.

Many researchers have summarized the criteria for judging masonry structures’ damage forms and grades according to numerous engineering experiences^8–11^. The criteria proposed by Burland et al.^8^ and Boscardin and Cording^9^ are widely used to judge the damage grades of masonry structures based on the crack width and maximum principal tensile strain.

Although the above principle is widely used due to its convenience, the influence of tunnel construction on adjacent buildings is a typical problem of tunnel soil–structure interaction. Many researchers have used numerical simulation for the analysis^12,13^. In some studies, buildings have been modeled as an elastic shell at the surface^14^, an equivalent beam^15^, or a bare frame^16^, which cannot effectively reflect the cracking characteristics of masonry structures. In some other analyses, masonry buildings have been modeled as a wall with discrete elements^17,18^ or a masonry façade wall^19,20^. The discrete element (DE) method can model bricks and mortar separately. This approach is more refined, but the complexity has also significantly increased. Masonry buildings are greatly simplified in the latter analysis method, referred to as the equivalent material approach. Masonry structures are regarded as a homogeneous material at the macro level and define anisotropy through micro material paraments^21,22^. Related research focuses on the direct relationship between different bricks, the influence of mortar discontinuity, and tensile strength. Some researchers have investigated the relationship between the compressive and tensile strength of masonry structures, mortar, and overall strength^23–25^. However, these studies have mainly concentrated on the materials, and the cracking phenomenon caused by the interaction between soil, the building, and its foundation is seldom considered. Giardina^26^ presented an experiment on a 1/10th scaled masonry façade subject to tunnel-induced settlements. A rubber cushion layer has regarded as the interaction between building and soil with no-tension behavior. The development law of cracks in the wall with openings has been examined. The study has mainly been based on experiments, rather than related to practical projects, and the differences are not investigated. Therefore, investigating the crack development law and damage grade of masonry buildings during shield tunneling is significant in practical engineering.

This study conducts cracking and damage analysis of masonry structures based on the jointed masonry model (JMM) caused by shield tunneling undercrossing with an oblique angle. Further, it analyzes the variation law of apparent damage of masonry buildings with shield tunneling and compares it with the current related evaluation standards of building damage. Thus, a rational judgment method based on the finite element calculation results has been proposed in this paper.

## Project background

**Figure 1:**
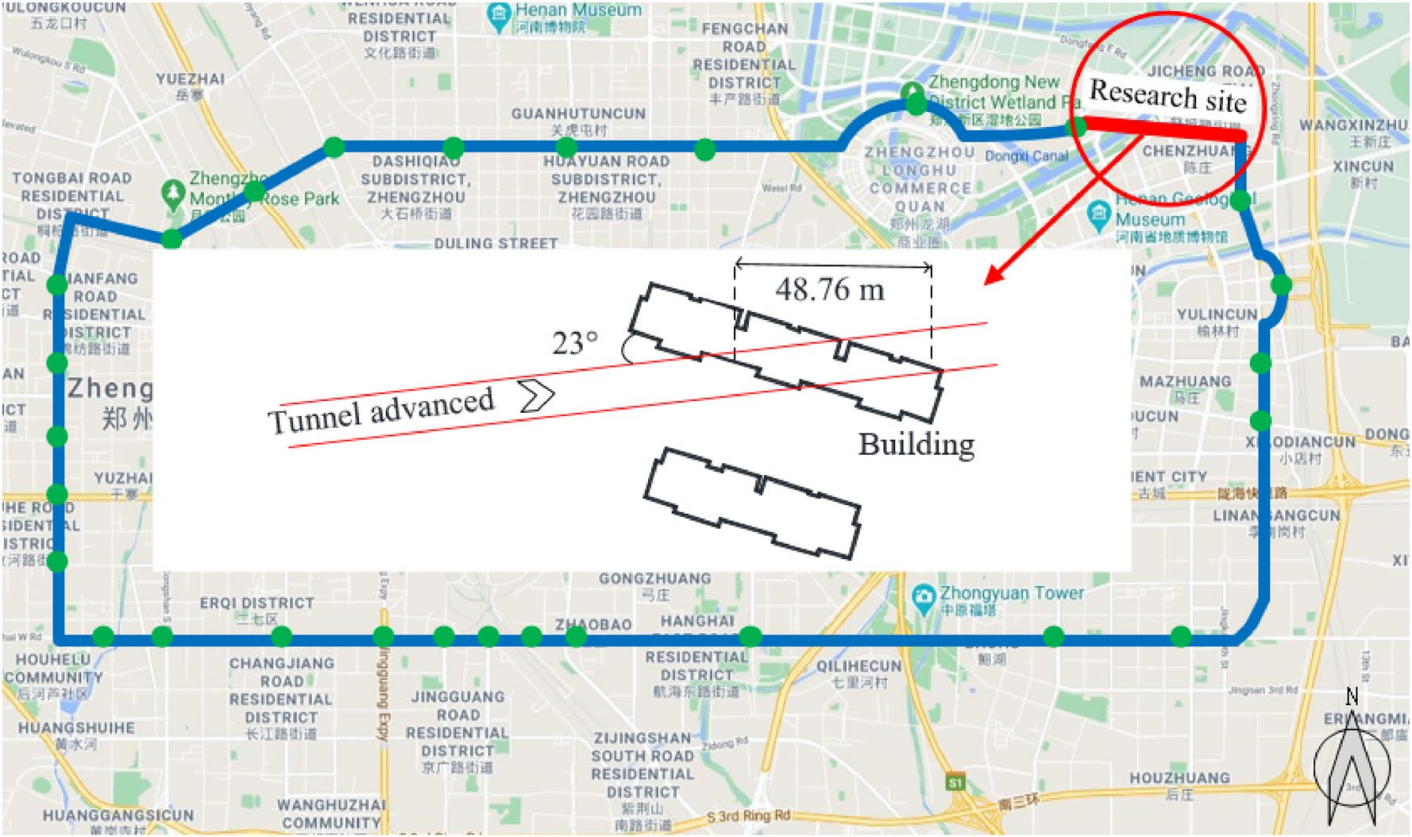
Location of the research site (China Baidumap, Version number: 18.8.0. link:https://map.baidu.com/zt/client/copyrightPc/index.html; Annotated by the author).

The research site is located in Zhengzhou City, Henan Province, in the middle of China, and is a part of Zhengzhou Metro Line 5, which is about 1500 m long. Zhengzhou borders the Yellow River to the north, mountainous and hilly areas to the west and southwest, and alluvial and diluvial plains of the Yellow River to the east. The double-line, single-circle shield constructs Metro Line 5, the left line has been built first, and the right line will be constructed 45 days later. The shield tunnel on the left line obliquely crosses the northwest-southeast masonry structure at an angle of 23, and the length of the underpass section is about 48.76 m (Fig. 1). The outer and inner diameters of the shield are 6.2 and 5.5 m, respectively. Further, C50 concrete lining segments are used, with a ring width of 1.5 m, and the depth of the tunnel is from 13.0 to 15.4 m.

**Figure 2:**
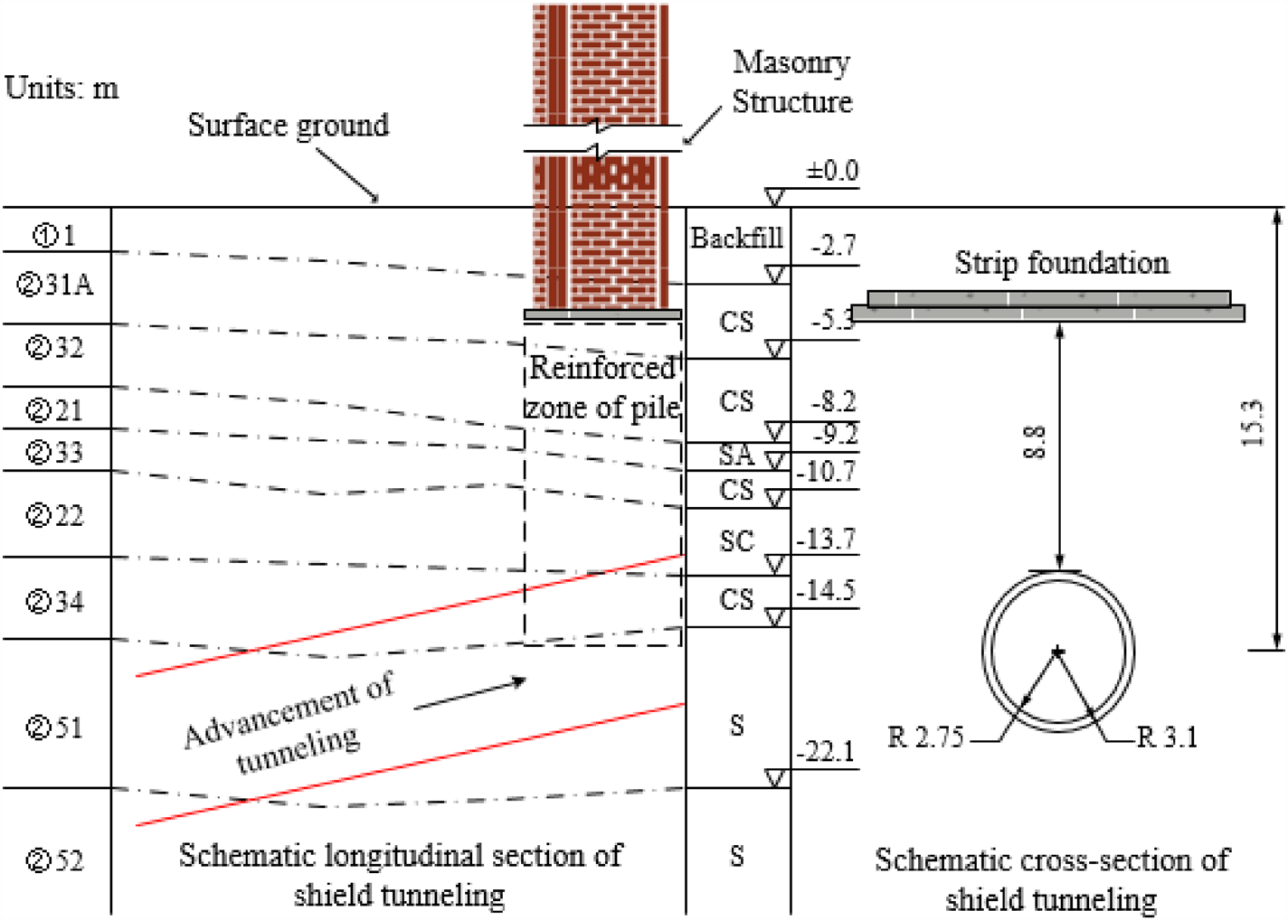
Relative position of the structure and tunnel.

The building is a six-story masonry structure with a length, width, and height of 75, 13, and 18.8 m, respectively. The C30 concrete strip foundation is adopted, with 500@950 cement deep mixing piles. During the shield tunnel construction, the number of cutter heads invading the cement–soil mixing piles is about 175, and the invading length is about 2.36–3.7 m. During the shield tunneling under-pass the masonry building, the maximum and minimum distance from the top of the tunnel to the ground are 13.2 m and 12.2 m, respectively. The relative position of the masonry structure and tunnel is shown in Fig. 2. The group CSC bottom of the masonry structure is mainly located in the ②51 fine sand layer, and the tunnel is located primarily on the ②51 silt soil layer. Table 1 presents the main physical and mechanical parameters of the soil.

**Table 1:** Soil property parameters.

Layer number	Gravity *γ* (kN/m^3^)	Compression modulus *E* (MPa)	Poisson ratio *v*	Cohesion *C* (kN/m^2^)	Friction angle *φ* (°)
①1 Artificial fill	18.0	4.0	0.33	16.0	26.0
②31A Clayey silt	18.9	9.2	0.30	20.0	22.1
②32 Clayey silt	19.4	8.5	0.30	19.9	21.2
②33 Clayey silt	19.9	7.5	0.32	19.0	20.4
②22 Silty clay	19.4	6.1	0.38	31.2	15.6
②34 Clayey silt	20.2	8.7	0.33	19.3	20.7
②51 Fine sand	19.4	20.0	0.27	1.5	32.0
②52 Fine sand	19.5	22.0	0.27	1.5	34.0

**Figure 3:**
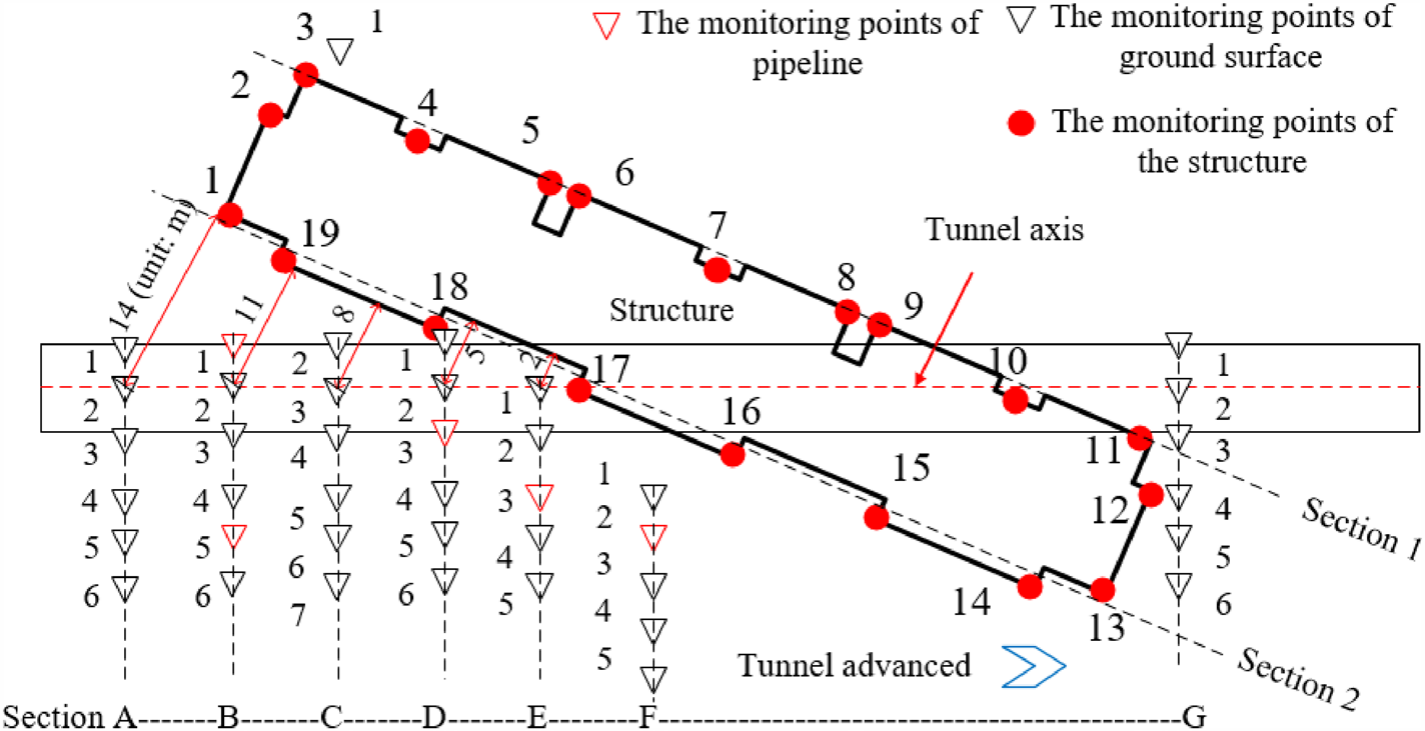
Layout of the monitoring points for the subsidence of the building and ground.

A series of settlement monitoring points were set up on the building and its surrounding surfaces to explore the tunneling influence. A total of 19 monitoring points of building settlement are located at the bottom corner around the building, and 41 monitoring points of the surface settlement are primarily arranged on the side of buildings due to the limitation of site conditions (Fig. 3).

### Finite element model

**Figure 4:**
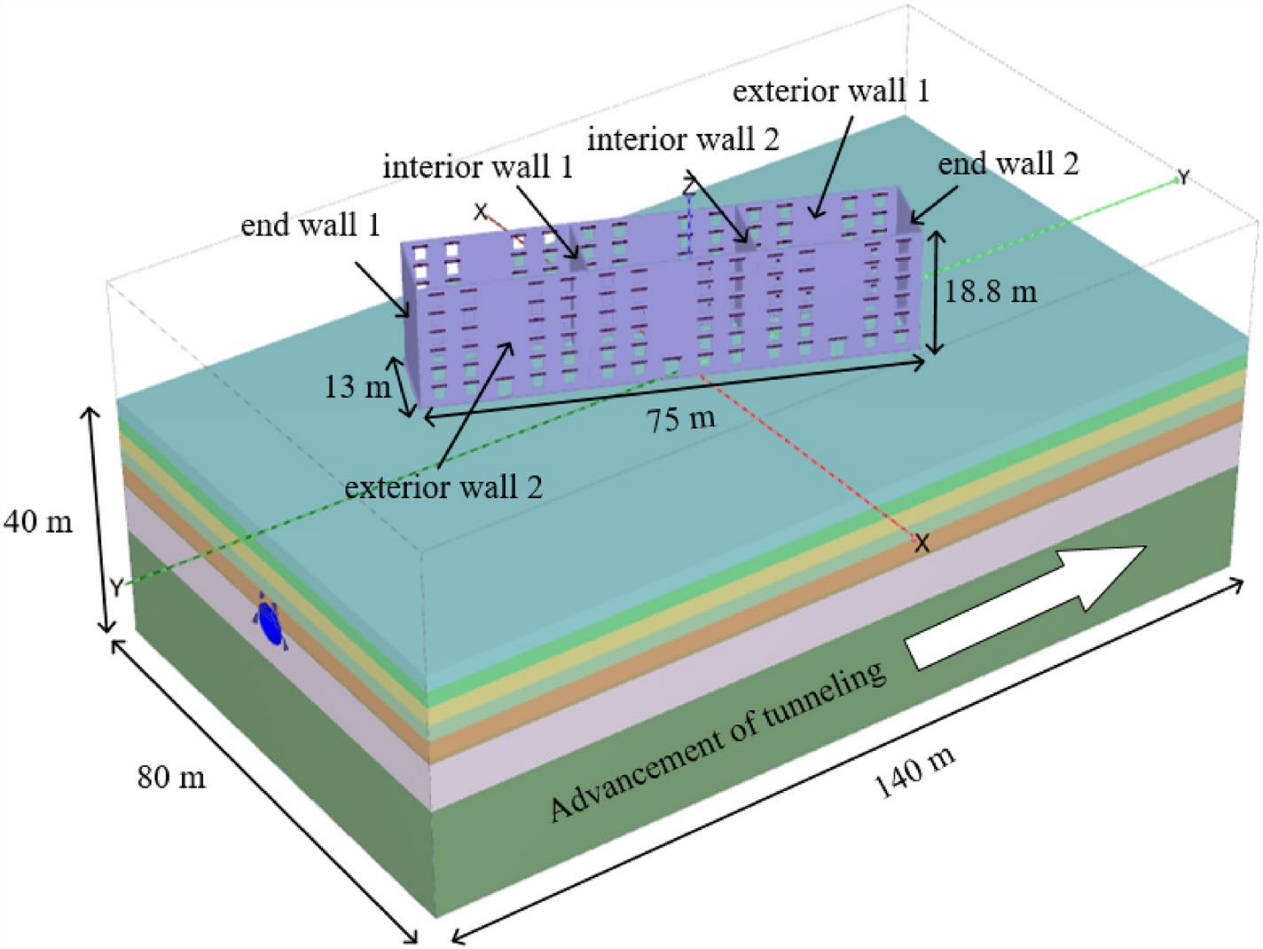
Finite element model.

Figure 4 shows the finite element model of the project. The model’s size is 80 m in width and 140 m long and is divided into 129,653 units and 212,719 nodes. A z-direction constraint is applied to the bottom of the model, and a horizontal constraint is applied to each side of the model. The building has been established for decades, hence the soil is considered to be consolidated.The initial stress of the soil is initialed according to the green field first and then the building add to the model with gravity and zero displacement. The coefficient of soil lateral pressure is *K*_0_ = 1 − sin*φ*′. Soil settlement caused by tunnel excavation is mainly controlled by tunnel volume loss, which will be explained in the following content.

#### Modeling of a masonry structure

**Figure 5:**
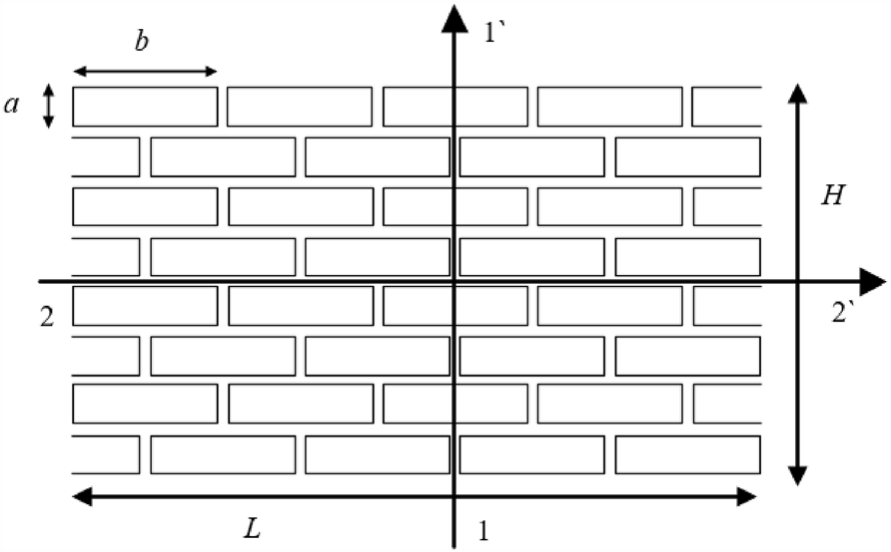
Definition of Orientations 1 and 2 in the plane.

The tensile and shear capacity of masonry structures are significantly weak. It often appears in cracks when the masonry structure is damaged, and the relationship between the crack and mortar direction is significant. However, the traditional linear elastic constitutive model cannot simulate this characteristic. Therefore, this study adopts the JMM, which has emerged in recent years to simulate buildings^21^. In this model, the masonry structure is described as a homogeneous anisotropic continuum at the macroscopic level. The mortar is simulated by the orthorhombic joints (Fig. 5). Since this paper is mainly aimed at brick masonry buildings, the parameters of this paper are obtained by testing with existing bricks^21^. For other forms of masonry structure, the model parameters can be obtained by testing or relevant literature.

According to homogenization theory, the macroscopic elastic and strength properties are derived^21^.

1 $$\frac{{v_{12} }}{{E_{1} }} = \frac{{v_{21} }}{{E_{2} }} = \frac{{\lambda_{b} }}{{2\left( {3\mu_{b} \lambda_{b} + 2\mu_{b}^{2} } \right)}}$$

2 $$\frac{{1}}{{E_{{1}} }} = \frac{4a}{{4abK_{n} + b^{2} K_{t} }} + \frac{1}{{4\mu_{b} }} + \frac{{\lambda_{b} + 2\mu_{b} }}{{4\left( {3\mu_{b} \lambda_{b} + 2\mu_{b}^{2} } \right)}}$$

3 $$\frac{{1}}{{E_{{2}} }} = \frac{{1}}{{aK_{n} }} + \frac{1}{{4\mu_{b} }} + \frac{{\lambda_{b} + 2\mu_{b} }}{{4\left( {3\mu_{b} \lambda_{b} + 2\mu_{b}^{2} } \right)}}$$

4 $$\frac{1}{G} = \frac{1}{{aK_{t} }} + \frac{4a}{{b^{2} K_{n} + 4abK_{t} }} + \frac{1}{{\mu_{b} }}$$

where *λ*_*b*_ and *μ*_*b*_ are Lame’s constants and *K*_*n*_ and *K*_*t*_ are the normal and shear joint stiffness, respectively.

The failure of the mortar joints is mainly affected by their tensile strength, cohesion, and friction with bricks. As shown in Fig. 5, the self-weight stress on the bottom of the masonry structure is more significant than that on the top, causing an interlocking effect of the bricks. This results in the strength of the bottom of the structure being more significant than that of the top. Therefore, the strength enhancement coefficient *β*, related to the brick size, is adopted in this model to consider this phenomenon. The ultimate value of *σ*_*t, i*_ with Mohr–Coulomb and tensile cutoff yield can be evaluated as

5 $$\sigma_{t,i} { = }\sigma_{t0,1} { + }\beta \sigma_{n,j} {\text{ + c}}_{0,j} \frac{\beta }{{\tan \varphi_{j} }}$$

6 $$\beta {\text{ = tan}}\varphi \frac{b}{2a}$$

**Figure 6:**
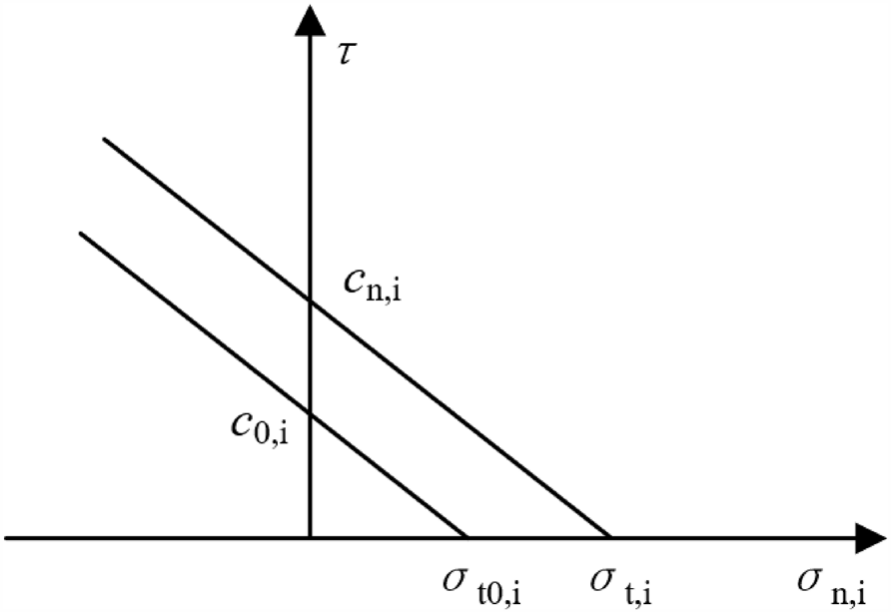
Strength envelope curve for joints (direction i = 1,2).

It can be characterized by translating the strength envelope curve along a direction parallel to the original one (Fig. 6).

The interlocking phenomenon only occurs in 2–2 directions. Thus, the strength development of the 1–1 direction can be ignored and evaluated as

7 $$\sigma_{{t,{2}}} { = }\sigma_{{t0,{2}}}$$

**Figure 7:**
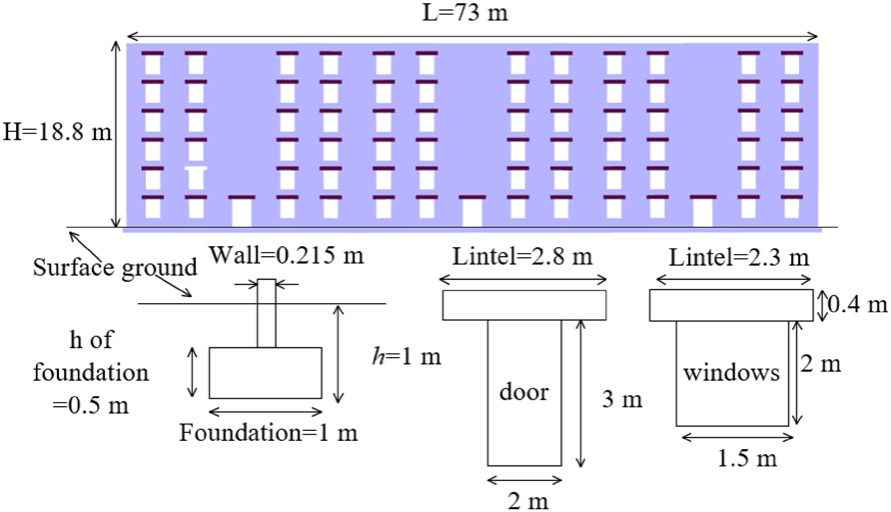
Masonry structure of the finite element model.

The effects of the presence of strip foundations have been taken into account in this paper's calculations (Fig. 7), considering that the floor and roof will not significantly affect the structural damage^27^, only the building’s exterior, end, and interior walls are considered for simplicity^19,26^. Table 2 summarizes the values of material parameters. The foundation in this project is a strip footing with a width of 1 m, a thickness of 0.5 m, and an embedded depth of 1 m. To avoid the collapse due to insufficient bearing capacity at the masonry wall opening and attempt to avoid the existence of “singularity” in the finite element mesh, the linear elastic material is used on the lintel of doors and windows. The geometric parameters of the lintel are set according to the architectural design drawing, and the elastic modulus and Poisson's ratio are set according to the parameters of the concrete C20. Table 3 summarizes the value of linear elastic materials parameters. Figure 7 shows the building size and related details.

**Table 2:** Physical and mechanical parameters of model masonry^22^.

Parameters	
Gravity *γ* (kN/m^3^)	15
Shear modulus *G* (kPa)	477,093
Poisson`s ratio *v*	0.12
Enhancement coefficient *β*	0.687
Cohesion *c* (kPa)	5
Distinguish mortar (joint) direction	
* α*_1_ (°)	90
* α*_2_ (°)	0
Friction angle *φ*_1-2_ (°)	31
Dilatancy angle *ψ*_1-2_ (°)	31
Tensile strength σ1–2 (kPa)	8.32

**Table 3:** Physical and mechanical parameters of the model elastic material.

Materials	Gravity*γ *(kN/m^3^)	Young’s modulus *E* (GPa)	Poisson’s ratio *v*
Lintel	25.0	25.5	0.2
Shield	40.0	210	0.08
Lining	25.0	32.5	0.2

#### Tunneling simulation

**Figure 8:**
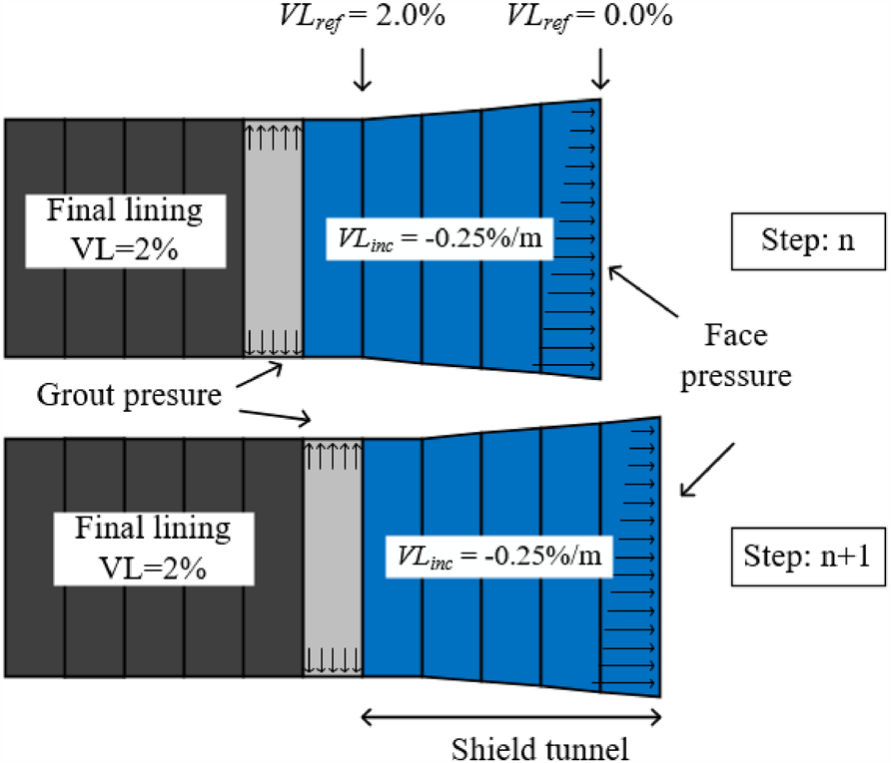
Tunnel boring process and the cone shape of the TBM.

In this study, the impact caused by the tunnel construction process is simulated using the “volume loss (VL) control” method^28^. The measured data define the grouting pressure, ground VL, face pressure, lining, and shield tunnel. The grouting pressure is 260 kPa at the tunnel crown, and the pressure increases linearly with a gradient of 14 kPa/m. The face pressure on the tunnel crown is 140 kPa with a gradient of 9 kPa/m. The excavation step is set to 2 m, and the shape of the shield machine is simulated by defining the shrinkage rate at different positions of the shield machine. The last ring is the tail of the shield that adopts a uniform shrinkage of 2.0%. Further, the remaining four rings adopt the linear shrinkage to simulate the shield machine’s shape with a linear contraction, with a reference value of 0.0% (*VL*_ref_) at the face with an increment of 0.25%/m (*VL*_inc_; Fig. 8). The distributed excavation method was adopted in the shield tunneling process^29^, with 37 steps and a 2-m excavation in each step (Fig. 8). The shield and lining adopt a suitable elastic constitutive model, stimulated by linear elastic plate elements. Table 3 summarizes the value of physical and mechanical parameters.

#### Volume loss

**Figure 9:**
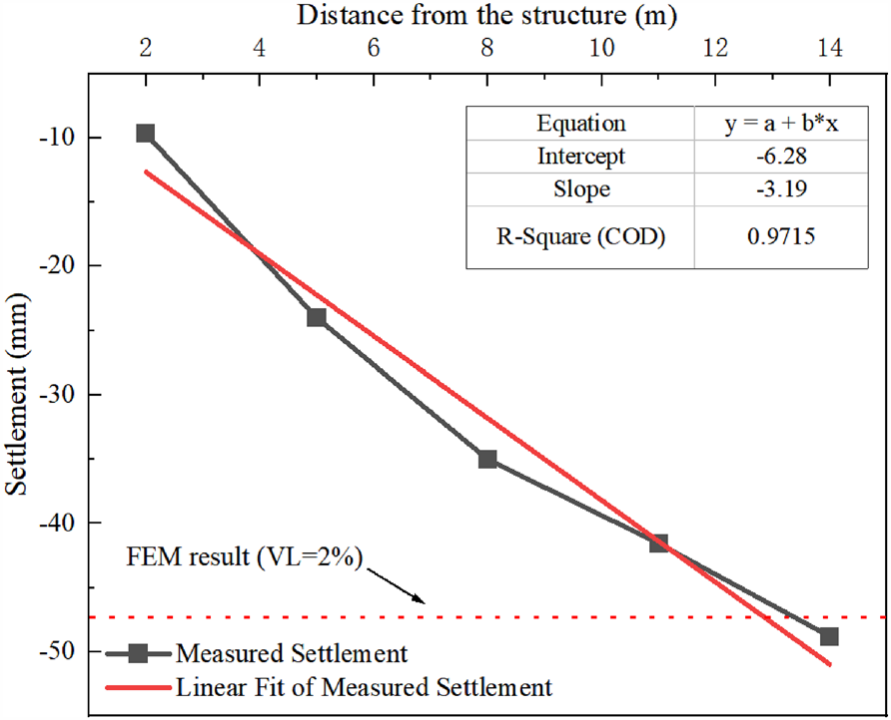
Relationship between surface measured settlement and the distance from the structure.

The VL induced by tunneling is the leading cause of building damage. In Fig. 9, the final settlement value of the surface ground monitoring point with the A–E section above the tunnel axis (A-2, B-2, C-3, D-2, and E-1) is drawn and compared with the finite element result directly above the tunnel axis. The settlement value of the surface ground increases with the distance from the building and gradually approaches the result of FEM, which has a uniform VL rate of 2%.

**Figure 10:**
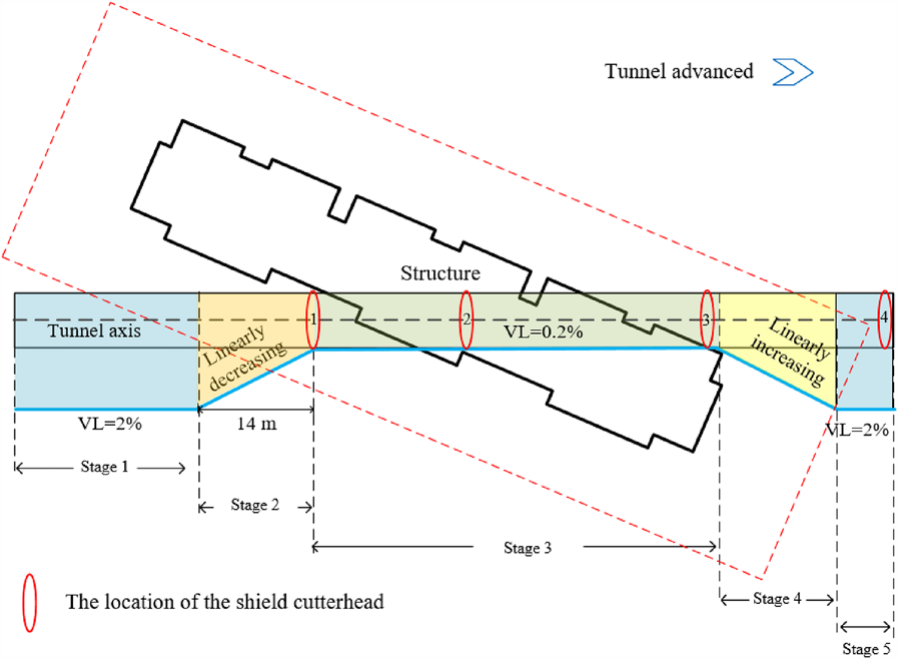
Schematic of the ground volume loss variation. (**a**) Section C. (**b**) Section G.

According to this phenomenon, the VL should be regulated by the distance from the structure. Considering the reinforcement effect of group foundation, VL is divided into five stages. The blue line indicates the control standard in Fig. 10. The construction process is divided into the following stages according to the relative position of the cutter head and building. (1) Stage 1: the shield cutter head is more than 14 m away from the building. (2) Stage 2: it is less than 14 m away from the building. (3) Stage 3: it is in the process of under-passing through the building. (4) Stage 4: it moves away from the rear side of the building. (5) Stage 5: it is far from the building.

## Results and discussion

The factors, including over-excavation and under-excavation, can induce the displacement of the ground, leading to the damage of buildings on the ground. Therefore, studying the damage to upper buildings is essential to ensure that the ground settlement is the same as the site’s working condition. Here, the correctness of the model is verified by comparing it with the measured ground settlement. The finite element method (FEM) results of masonry structures with cutter heads in front of and under the building and shield tails far from the building are taken for analysis.

### Ground settlement

**Figure 11:**
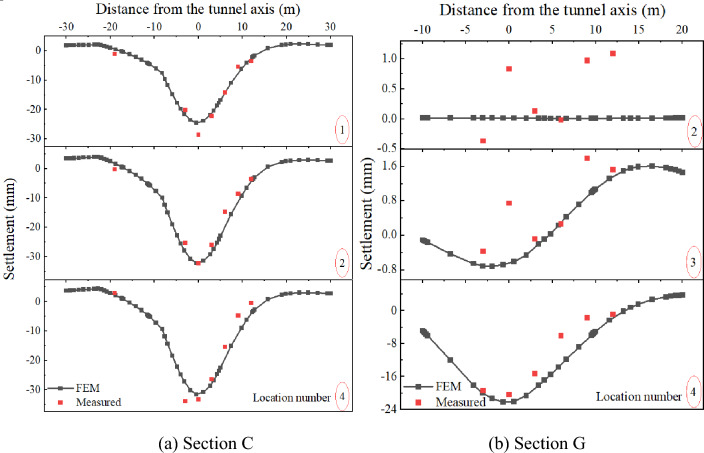
Comparison between the field measurement and FEM results of surface settlement. (**a**) Shield cutter head reaching the building. (**b**) Shield tunnel reaching the middle position of the building. (**c**) Shield tunnel far from the building.

To investigate the influence of tunneling on the surface settlement, typical sections (sections C and G, Fig. 3) were considered for the analysis. Figure 10 shows the set location of the cutter head according to the relative position of the shield cutter head and the building. Figure 11a shows that the FEM results and measured values exhibit appropriate consistency across different stages. As shown in Fig. 11b, the settlement value of Section G from location Nos. 2–3 was within 1 mm, accounting for about 5% of the total settlement value. However, from location Nos. 2–4, as the cutter head approaches the monitored section, the settlement value begins to fluctuate, the same as in general cognition. From the relative position of the section and building, Section G is closer to the building than Section C, confirming the above hypothesis. By comparing the result obtained using the FEM with the measured settlement, the FEM model can appropriately reflect the actual working condition.

### Cracking and damage analysis of exterior wall 1

**Figure 12:**
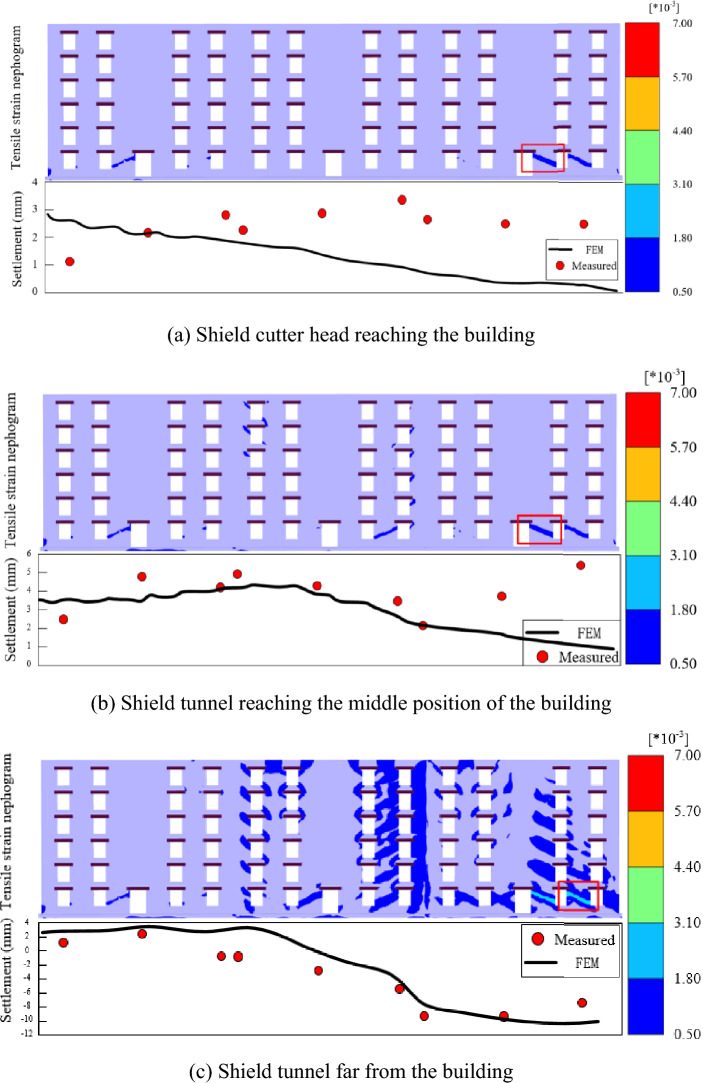
Tensile strain nephogram of exterior wall 1 at different stages. (**a**) Shield cutter head reaching the building. (**b**) Shield tunnel reaching the middle position of the building. (**c**) Shield tunnel far from the building.

The measured settlement of the building and FEM results were compared as shown in Fig. 12. Exterior wall 1 was in an uplift state before the shield machine leaves the building. When the shield machine is far from the building, the exterior wall 1 changed to settlement. The coincidence between the FEM and measurement results gradually increases with shield tunneling.

As the tensile strength of masonry is influenced by many factors (e.g., building materials and composition), the ultimate tensile strain value of masonry while cracking is not constant. In this study, 0.05% is selected as the critical tensile strain value (Table 4). The minimum value of the legend is set to 0.05%, and the tensile strain area displayed on the wall is the area where the building damaged.

**Table 4:** Critical strains for different structures and materials.

Researchers	Tensile strain (%)	Structure type
Polshin and Tokar (1957)	0.05	Brick and concrete structure
Burhose (1969)	0.038–0.06	Brick wall
Mainstone (1971, 1974)	0.02–0.137	Frame structure with brick
Littlejohn (1974)	0.02–0.03	Brick wall
Burland and Worth (1974)	0.03–0.09	Wall and slab
Boscardin and Cording (1989)	0.05	Masonry structure

Figure 12a shows the tensile strain of exterior wall 1 when the shield cutter head reaches the building (the cutter head is located at location No. 1). As shown in the box in Fig. 12a, the cracks only appear at the bottom of exterior wall 1, and the maximum tensile strain value occurs at the lower right side of the wall, which is 0.11%. The affected area begins to widen with the advancement of the shield. When the shield tunnel reaches the middle position in Stage 3 (the cutter head is located at location No. 2), the maximum tensile strain value is 0.14%, which is only 0.03% higher than that of the previous stage. As shown in Fig. 12b, the area where the maximum tensile strain occurs is the same as in the last stage. The state of exterior wall 1 changes from uplift to settlement when the shield tunnel is far from the building, and the crack development area increases sharply. As shown in the box in Fig. 12c, the maximum tensile strain value increased to 0.348%, and the position shifted to the right.

### Cracking and damage analysis of exterior wall 2

**Figure 13:**
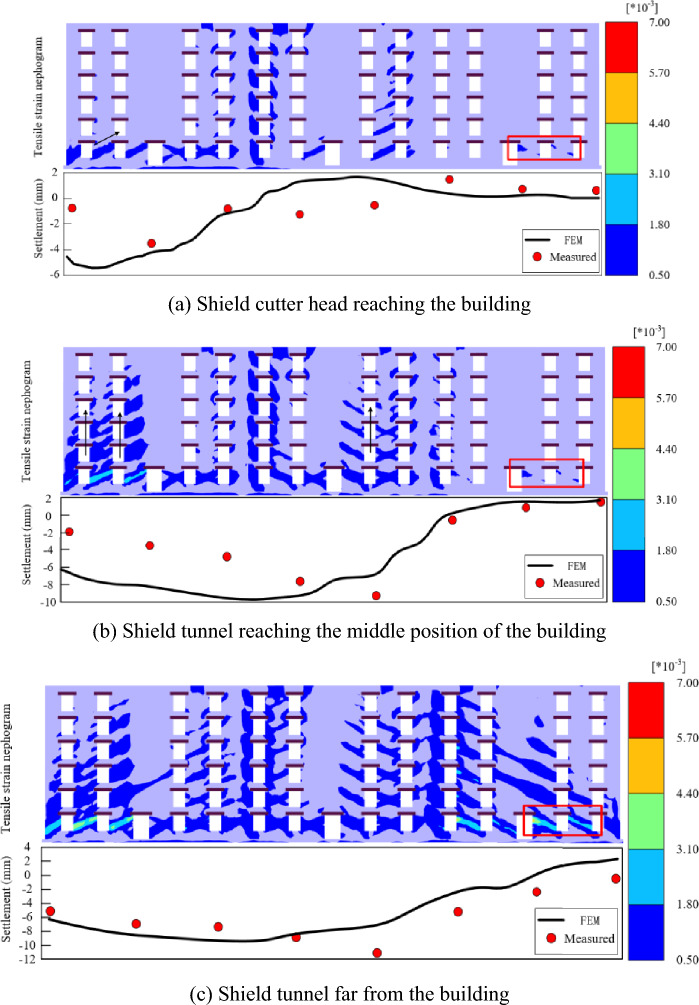
Tensile strain of exterior wall 2 at different stages. (**a**) Shield cutter head reaching the building. (**b**) Shield tunnel reaching the middle position of the building. (**c**) Shield tunnel far from the building.

Figure 13 shows the tensile strain of exterior wall 2 at the different stages. The left side of exterior wall 2 is located in the main settlement area, whereas the right side is slightly uplifted when the shield cutter head reaches the building. At this time, the cracks development area is mainly on the left side of the wall and a small area on the right side. As shown in Fig. 13a, the cracks occur at the corners of doors and windows. With the shield tunneling, the cracks began to develop from the bottom of the wall to the top of the previous cracks. The crack development area on the right side of the wall started to increase, with the concentrated settlement area moved to the right. However, compared with the previous stage, the crack position on the right side of the building did not change significantly (Fig. 13b). The maximum value of settlement occurs at the intersection of the building and tunnel when the shield tunnel is far from the building. Further, the cracks will be distributed symmetrically in the shape of “^” on both sides of the intersection. The direction where the cracks spread is the same with the settlement curve, and the crack extending direction is orthogonal to the curve. The maximum tensile strain value of exterior wall 2 increased from 0.26 to 0.62%, and the location where the maximum crack has shifted to the right side of the wall. As shown in Fig. 13c, the maximum crack was located at the original tiny crack, further developed in the previous stage.

### Cracking and damage analysis of the end and interior walls

**Figure 14:**
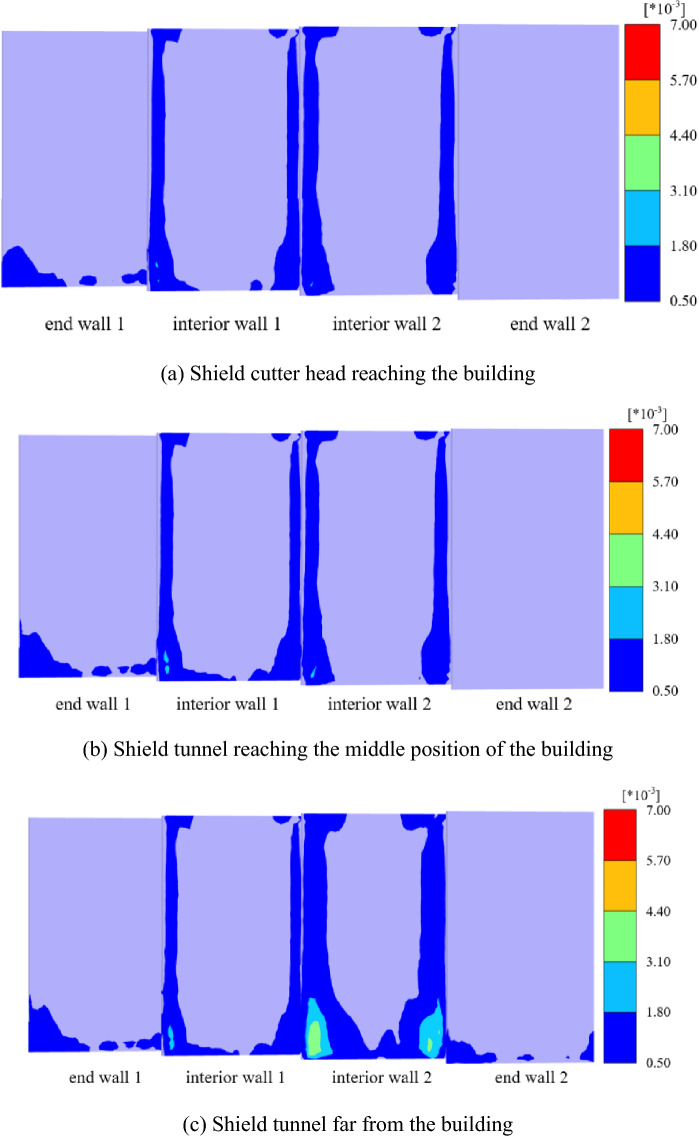
Tensile strain nephogram of the interior and end walls at different stages.

Figure 14 shows the tensile strain of the end and interior walls at different stages. The impact of the end walls on both sides of the building is small. The cracks mainly occur in the interior wall and are close to exterior wall 2. The crack area of the end wall increases slightly with shield tunneling. However, the interior wall’s tensile strain value and area change dynamically during shield crossing, which may be related to the wall being closer to the tunnel axis. The maximum tensile strain value also increases from 0.21 to 0.36% with shield tunneling, and the location where the maximum crack shifts from interior wall 1 to interior wall 2. As indicated in the FEM results of the overall tensile strain of the building, the maximum crack occurs on exterior wall 2 when the shield tunnel is far from the building, and the tensile strain decreases gradually along exterior wall 2 (EW2), interior wall (IW), and exterior wall 1 (EW1).

### Damage degree analysis of masonry structures

Combined with the masonry damage judgment standard of Burland et al.^8^ and Boscardin and Cording^9^, Netzel^10^ proposed the corresponding relationship between crack width and maximum principal tensile strain value (Table 5). As indicated in the results of FEM in the final stage, the maximum tensile strain value of the masonry structure has reached 0.62%. This implies that the building has already suffered severe damage as per the judgment standard of building damage grade presented in Table 5. However, from the in situ test results, the building on the site is far from severe damage.The FEM tensile strain results indicate that only a few areas have higher tensile strain values. Hills^30^ deems that the corner of doors and windows will affect the stress calculation results. Yiu^27^ suggested that should apply denser grids in the intersection area where the opening area of doors and windows, leading to stress concentration. Yiu^27^ highlighted that the maximum tensile strain often only accounts for a very small part, so the concept of “characteristic tensile strain = *ηA*_wall_” was proposed, where *A*_wall_ is the total area of the wall being assessed. It determined a value that can include the entire *η* times area as the characteristic strain value while ignoring the tensile strain value with a relatively small area due to grid quality problems. Note that the value *η* = 0.99 taken by Yiu is arbitrary.

**Table 5:** Judgment standard of the building damage grade^10^.

Category of damage	Damage level	Description of typical damage and ease of repair	Approximate crack width (mm)	Limiting tensile strain levels (%)
Aesthetic damage	Negligible	Hairline cracks of less than about 0, 1 mm width	Up to 0, 1 mm	0–0.05
	Very slight	Fine cracks can easily be treated during normal decoration	Up to 1 mm	0.05–0.075
	Slight	Cracks can easily be treated during normal decoration, and some cracks may be required waterproofing	Up to 5 mm	0.075–0.15
Functional damage	Moderate	Cracks may need to be patched with bricks. Service pipes may fracture. Poor impermeability	5–15 mm or a number of cracks > 3 mm	0.15–0.3
	Severe	The whole wall needs to be repaired, such as replacing part of the wall. The floor and wall are inclined, and the pipeline is damaged	15–25 mm, but also depends on the number of cracks	> 0.3
Structure damage	Very severe	The building is unstable and needs to be repaired	Usually > 25 mm, but depends on the number of cracks	

**Figure 15:**
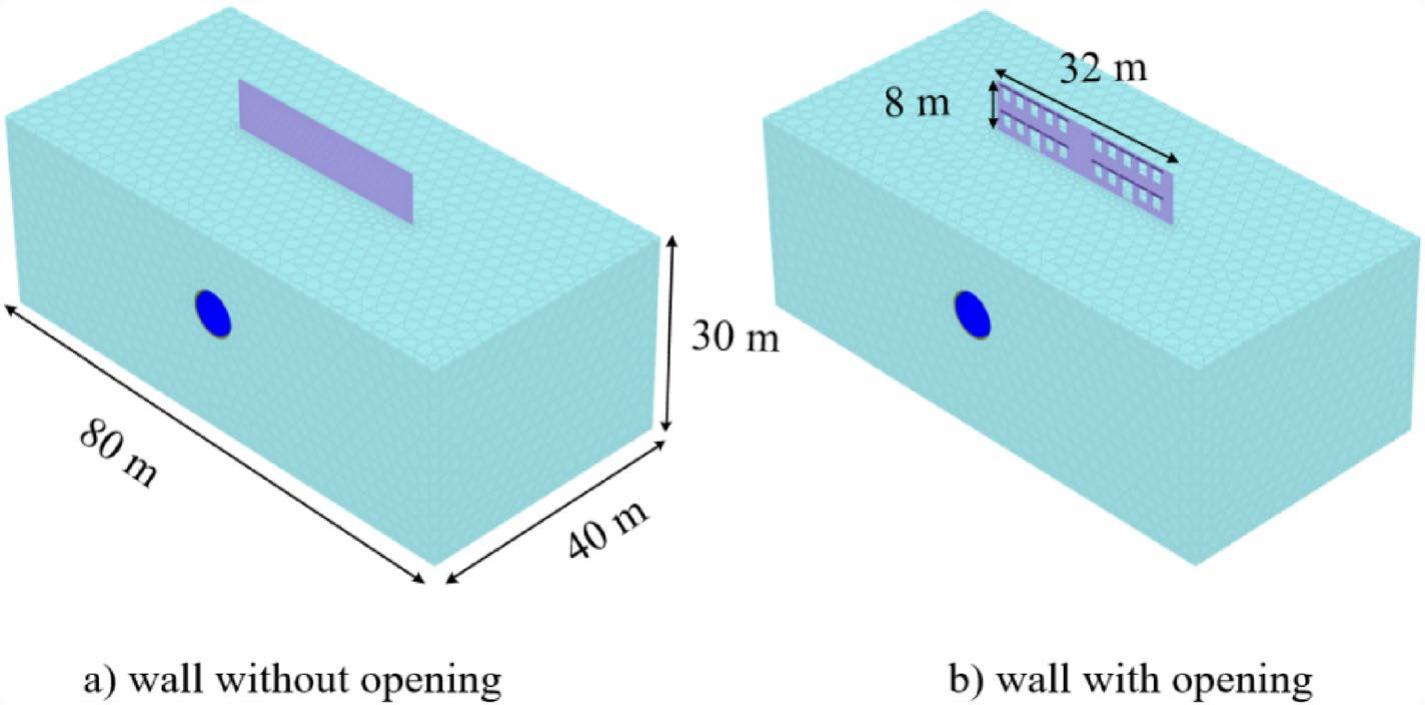
Finite element model with the wall.

**Figure 16:**
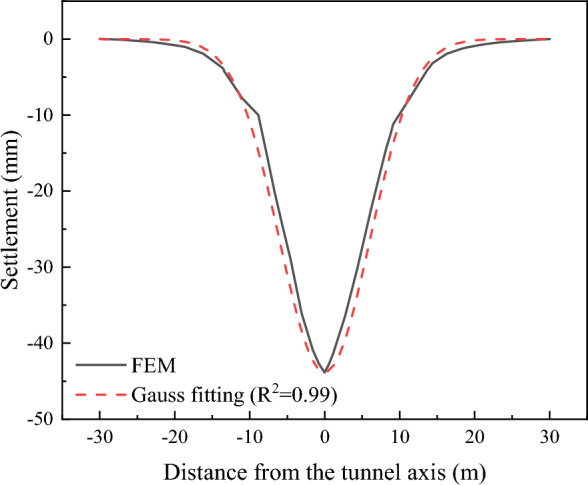
Ground surface settlement curve.

**Figure 17:**
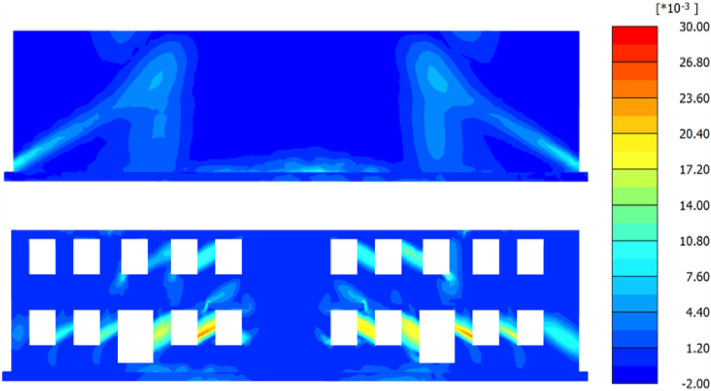
Tensile strain nephogram of the wall.

To encounter the gap between the JMM and theoretical calculation, the finite element models of the wall with and without openings are established. The ultra-fine mesh is used in FEM (Fig. 15). According to the structure size used in the FEM, the maximum tensile strain value is calculated using the limit tensile strain method (LTSM), initiated by the work of Burland et al.^8^ and Boscardin et al.^9^, and compared with FEM results. The surface settlement used in the LTSM is obtained by Gauss fitting from the FEM results (Fig. 16). Figure 17 shows the nephogram of the maximum principal tensile strain of the wall calculated by FEM. The crack development was consistent with the previous calculation results, and no evident cracks at the maximum settlement exist. The maximum tensile strain of open walls and without open walls is 1.04% and 2.57%, respectively. Therefore, the JMM can reflect the stress concentration phenomenon at the opening area. However, it will lead to a significant increase in the calculation results of the maximum tensile strain value. The result calculated using the LTSM is significantly less than the FEM results. Compared with the judgment standard of building damage grade presented in Table 5, the theoretical result judges that the wall is in a medium damage state. In contrast, the finite element results significantly exceed the severe grade.

**Figure 18:**
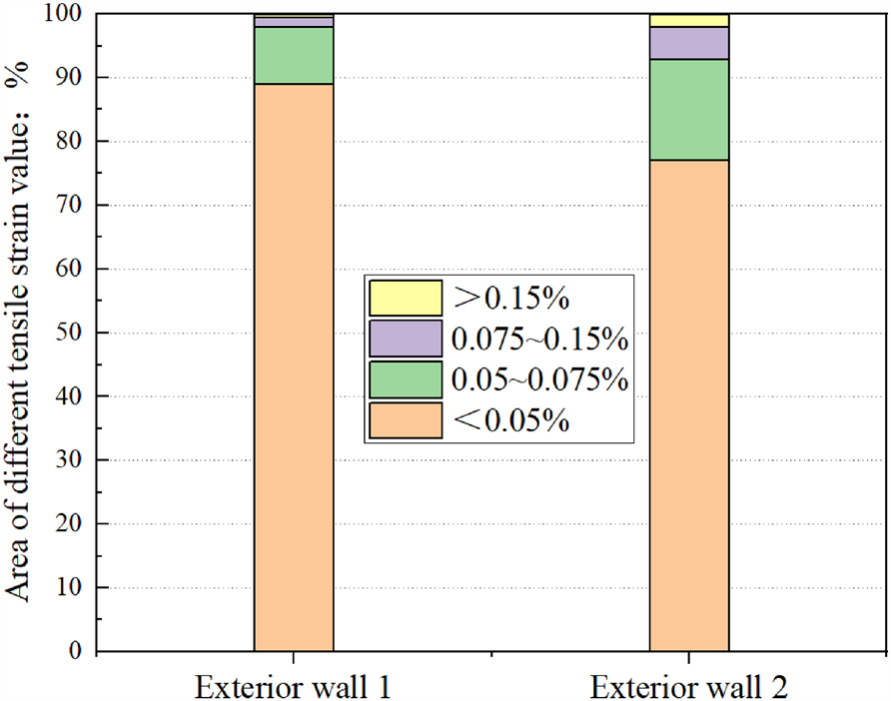
Wall damage bar chart.

As indicated in the results of the tensile strain nephogram, the JMM can well reflect the cracking characteristics of masonry structures. However, it is quite conservative to judge the damage degree of the building based on the tensile strain value. This paper divides the nephogram of tensile strain after the shield is far from the building into four damage levels. The total area of the building facade occupied by the four tensile strain areas is compared (Fig. 18). The area of exterior wall 1 with a tensile strain value of 0.05% accounts for 11% of the total area. However, the area with a tensile strain value of 0.15% accounts for less than 1% of the entire area. Although its maximum tensile strain was 0.348%, it could not reflect the actual damage to the building. The proportion of the total area with a tensile strain value greater than 0.05% of exterior wall 2 reaches 23%. As shown in Fig. 15, with an increase of the area proportion of 0.05–0.075% level of the total area, the areas with 0.075–0.15% level and higher than 0.15% level tensile strain also increase. The maximum principal tensile strain value reaches 0.62% in the final stage, and many areas have a tensile strain value greater than 0.075%. As indicated in the area proportion of different tensile strain areas and the judgment standard presented in Table 5, the damage grade of exterior wall 1 should be slight, whereas the damage grade of exterior wall 2 should be moderate.

## Conclusions

This study focused on cracking and damage analysis of a masonry structure based on Joint Masonry Model caused by a obliquely undercrossing tunnel. The damage and cracks of masonry structures due to shield tunneling at different stages were investigated. The following are the main conclusions:

1. Compared with the theoretical calculation method LSTM, the method provide by this paper can give the detail damage location of the masonry structure caused by shield construction. Due to the existence of door and window openings, the result of JMM is larger than LSTM result, the differences can be modified by concept of “characteristic tensile strain”.
2. The wall of the masonry building encounter the shield face first suffers more damage than the later ones. According to JMM result in this study, external wall 1 is in a slightly damaged state, whereas exterior wall 2 is in a moderately damaged state.
3. At the beginning of tunnlling, the damage were generated from a small area at the bottom for wall 1, but a larger area at the top of the building for wall 2. When tunneling to the middle of the building, the area increase more at the top of the building, but the maximum damage occurred at the bottom of the building. After tunneling, the damage area are mostly above the bottom of the building, but the maximum damage occurred at the first floor of two sides.

## Data Availability

Data analysed in this study are available upon reasonable request from the corresponding author (Jian Chen).

## References

[1] Son M, Cording EJ (2005). Estimation of building damage due to excavation-induced ground movements. J. Geotech. Geoenviron. Eng..

[2] Chakeri H, Ozcelik Y, Unver B (2013). Effects of important factors on surface settlement prediction for metro tunnel excavated by EPB. Tunn. Undergr. Sp. Technol..

[3] Boldini D, Losacco N, Bertolin S, Amorosi A (2018). Finite element modelling of tunnellin-induced displacements on framed structures. Tunn. Undergr. Sp. Technol..

[4] Mroueh H, Shahrour I (2003). A full 3-D finite element analysis of tunnelling—adjacent structures interaction. Comput. Geotech..

[5] Camós C, Molins C (2015). 3D analytical prediction of building damage due to ground subsidence produced by tunneling. Tunn. Undergr. Sp. Technol..

[6] Dimmock PS, Mair RJ (2008). Effect of building stiffness on tunnelling-induced ground movement. Tunn. Undergr. Sp. Technol..

[7] El Kahi E, Deck O, Khouri M, Mehdizadeh R, Rahme P (2020). A new simplified meta-model to evaluate the transmission of ground movements to structures integrating the elastoplastic soil behavior. Structures.

[8] Burland, J. B., Broms, B. B., & de Mello,V. F. B. Behavior of foundations and structures. State-of-the-art report. In *Proc.9th lnt.Conference on Soil Mechanics and Foundation Engineering*, Vol. 2,495–546 (Japanese Society of Soil Mechanics and Foundation Engineering, 1977).

[9] Boscardin MD, Cording EJ (1989). Building response to excavation-induced settlement. J. Geotech. Eng..

[10] Netzel HD (2009). Building Response Due to Ground Movements.

[11] Skempton AW, MacDonald DH (1956). The allowable settlement of buildings. Proc. Inst. Civil Eng..

[12] Bilotta E, Paolillo A, Russo G, Aversa S (2017). Displacements induced by tunnelling under a historical building. Tunn. Undergr. Sp. Technol..

[13] Fu J, Yu Z, Wang S, Yang J (2018). Numerical analysis of framed building response to tunnelling induced ground movements. Eng. Struct..

[14] Namazi E, Mohamad H (2013). Assessment of building damage induced by three-dimensional ground movements. J. Geotech. Geoenviron. Eng..

[15] Zhao C, Schmüdderich C, Barciaga T, Röchter L (2019). Response of building to shallow tunnel excavation in different types of soil. Comput. Geotech..

[16] Khabbaz H, Gibson R, Fatahi B (2019). Effect of constructing twin tunnels under a building supported by pile foundations in the Sydney central business district. Undergr. Sp..

[17] Parisi F, Lignola GP, Augenti N, Prota A, Manfredi G (2011). Nonlinear behavior of a masonry sub-assemblage before and after strengthening with inorganic matrix-grid composites. J. Compos. Constr..

[18] Lemos JV (2007). Discrete element modelling of masonry structures. Int. J. Arch. Herit..

[19] Burd H, Houlsby G, Augarde C (2000). Modelling tunnel-induced settlement of masonry buildings. Proc. Inst. Civil Eng. Geotech. Eng..

[20] Ceradini, V. Modellazione e sperimentazione per lo studio della struttura muraria storica. Ph.D. Thesis, University Rome ‘La Sapienza’ **[in Italian]** (1992).

[21] Massart TJ, Peerlings RHJ, Geers MGD (2007). Structural damage analysis of masonry walls using computational homogenization. Int. J. Damage Mech.

[22] De Felice G, Amorosi A, Malena M (2010). Elasto-plastic analysis of block structures through a homogenization method. Int. J. Numer. Anal. Methods Geomech..

[23] Wanda GL, Amorosi A, Boldini D (2019). Jointed masonry model A constitutive law for 3D soil-structure interaction analysis. Eng. Struct..

[24] Borri A, Corradi M, Galano L, Vignoli A (2004). Analisi Sperimentali e numeriche per la valutazione della resistenza a taglio delle murature. Ingegneria Sismica.

[25] Restrepo LF, Magenes G, Griffith MC (2014). Dry stone masonry walls in bending Part I: Static tests. Int. J. Arch. Herit..

[26] Giardina G, Alessandra M, Hendriks MAN (2012). Experimental analysis of a masonry façade subject to tunnelling-induced settlement. Eng. Struct..

[27] Yiu WN, Burd HJ, Martin CM (2017). Finite-element modelling for the assessment of tunnel-induced damage to a masonry building. Géotechnique.

[28] Chen R (2016). Investigation of response of metro tunnels due to adjacent large excavation and protective measures in soft soils. Tunnell. Undergr. Sp. Technol..

[29] Meng F (2018). Effects of tunneling-induced soil disturbance on the post-construction settlement in structured soft soils. Tunn. Undergr. Sp. Technol..

[30] Hills D (2011). Characteristics of the process zone at sharp notch roots. Int. J. Solids Struct.

